# Eyelid-associated complications after autogenous fat injection for cosmetic forehead augmentation

**DOI:** 10.1186/1471-2415-13-32

**Published:** 2013-07-10

**Authors:** Ji-Sun Paik, Won-Kyung Cho, Gyeong-Sin Park, Suk-Woo Yang

**Affiliations:** 1 Department of Ophthalmology and Visual Science, College of Medicine, Seoul St. Mary’s Hospital, The Catholic University of Korea, Seoul, Korea; 2 Deparment of Ophthalmology and Visual Science, College of Medicine, Daejeon St. Mary’s Hospital, The Catholic University of Korea, Seoul, Korea; 3 Department of Pathology, College of Medicine, Seoul St. Mary’s Hospital, The Catholic University of Korea, Seoul, Korea

**Keywords:** Autogenous fat injection, Lipogranuloma, Eyelid swelling, Eyelid lumps

## Abstract

**Background:**

We report two cases of unilateral upper eyelid swelling with multiple small lumps as an unusual complication of autogenous fat injection for cosmetic forehead augmentation.

**Case presentation:**

Two female patients were referred to our clinic for unusual unilateral eyelid swelling, with multiple small lumps. The duration of symptoms differed in each case, but both patients had a history of autogenous fat injection for cosmetic forehead augmentation at a local plastic surgery clinic. The lumps were small (diameter 5 mm~10 mm), palpable, hard, and nonmobile, and were evaluated by magnetic resonance imaging (MRI). Lumps from the eyelids of two patients were excised under general anesthesia. All of the masses were located deeply and found near the superior orbital rim or lateral orbital rim. The lumps exhibited chronic inflammation with fibrosis. Some of the lumps showed foamy histiocytic aggregation and foreign body lipogranuloma, resulting from iatrogenic fat injection. After excision, all masses and swelling disappeared, and moderate ptotic eyelid or lagophthalmos of affected eyes also improved.

**Conclusions:**

To our knowledge, eyelid swelling with multiple lumps in the eyelid is a very rare complication of autogenous fat injection for cosmetic forehead augmentation. This report should be helpful for ophthalmic clinicians who encounter these unusual symptoms.

## Background

Autogenous fat is an easily accessible, renewable resource that can be harvested from multiple sites with little or no morbidity. After transfer to other locations, autogenous fat serves as non-allergenic, well-tolerated, supple, and versatile implant material. Therefore, autogenous fat injection into the periorbital or mid-face region is a common type of cosmetic surgery for rejuvenation in middle-aged and older Western subjects. In Asians, autogenous fat injection has become increasingly popular for forehead augmentation in relatively young people to enhance the esthetics of the typical flat Asian forehead. Although autogenous fat has good long-term safety and no severe adverse reactions, we encountered a case series of eyelid swelling with multiple mass-like lesions after dermal injection of autogenous fat. These complications are reported in order to assist clinicians in future diagnoses of patients after autogenous fat injection.

## Case presentation

### Case 1

**Figure 1:**
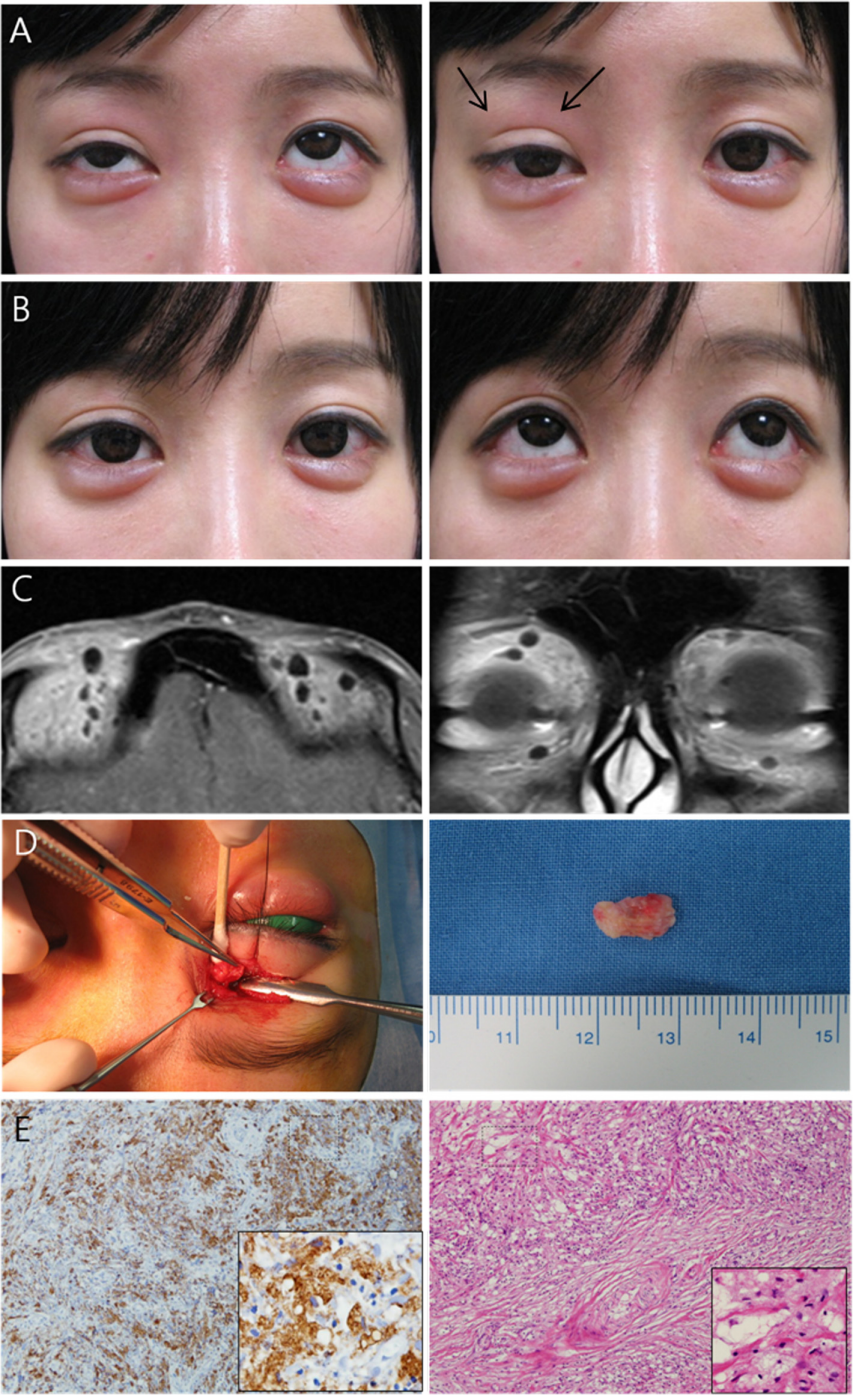
**Case 1. A)** Preoperatively, this patient had mild eyelid swelling with moderate blepharoptosis and multiple small nodules palpable deeply around the medial superior orbital rim. **B)** One month postoperatively, the masses and eyelid swelling had disappeared, and the blepharoptosis had been corrected. **C)** Preoperative MRI scanning. **D)** A photograph during surgery. The largest excised mass consisted of granulation tissue measuring 10× 6 mm. **E)** Microscopically, the specimen contained chronic inflammatory cells with fibrohistiocytic proliferation surrounded by amorphous collagenous material (H&E stain, ×100, ×400 with magnification). Histiocytes were positive for CD68 (×100, ×400 with magnification).

A 26-year-old woman complained of eyelid swelling and blepharoptosis of the right upper lid for 1 month that had developed slowly over the previous 6 months. On examination, a small mass 2~3 mm in diameter was seen laterally and another small mass measuring 5~7 mm in diameter was seen medially; these masses were not easily movable and poorly demarcated. A medially placed mass was palpated deeply near the superior orbital rim. No tenderness or skin discoloration was noted. Moderate blepharoptosis of the right upper lid was observed (Figure 1A). Magnetic resonance imaging (MRI) with enhancement revealed a diffuse enhancing mass that was larger than the clinically palpated mass (Figure 1C). On further questioning, the patient reported that she had twice received autogenous fat injection for forehead augmentation at a clinic. The first injection used fresh autogenous fat from her thighs and the second injection used previously harvested fat that had been stored frozen for one month after the first injection. She had undergone upper eyelid blepharoplasty via an incision 7 years earlier. Therefore, we planned a surgical excision using an eyelid crease skin incision in the upper eyelid. During the surgical procedure, the tissue between the orbital septum and preaponeurotic fat was gently dissected. Thick adhesions and fibrosis were removed, and gray-pink colored masses were found near the superior orbital rim. During excision, grayish jelly-like material was found around and within the masses. The masses did not have distinct boundaries and were not easily separated and removed (Figure 1D), because they adhered firmly to the surrounding structures. Microscopic examination of the specimen showed marked chronic inflammation with pseudogranulomatous foamy histiocytic aggregation, which was confirmed to be a lipid-phagocytic reaction or oil-associated foreign body reaction and fat necrosis (Figure 1E). Blepharoptosis and swelling occurred immediately after surgery, but 1 month later, the blepharoptosis and swelling had disappeared almost completely (Figure 1B). No masses were seen 18 months after surgery.

### Case 2

**Figure 2:**
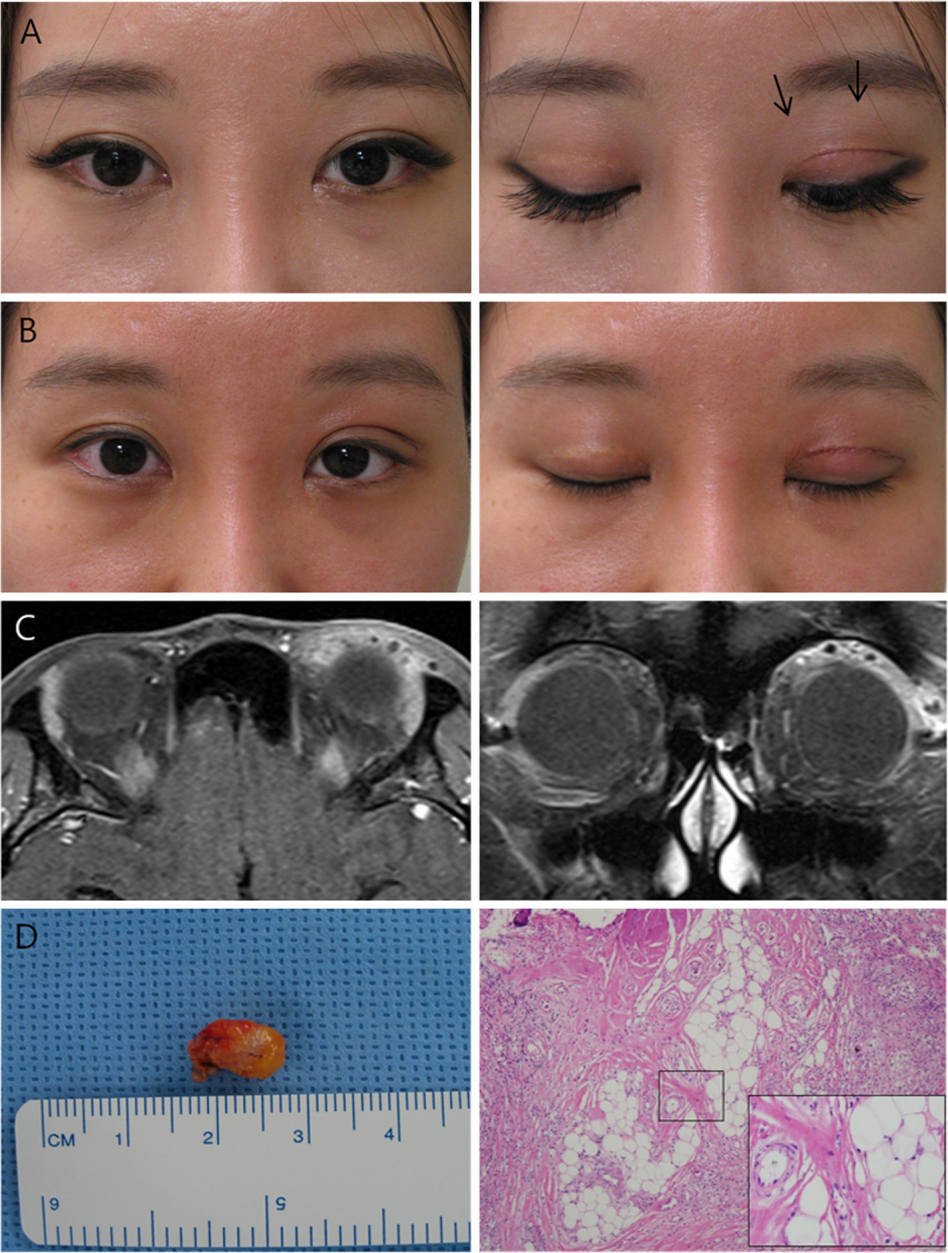
**Case 2. A)** Preoperatively, this patient had small nodoular masses with mild lagophtlmos. **B)** Two months postoperatively, no mass remained and mild lagophthalmos was improved. **C)** Coronal and axial enhanced MRI views showing diffuse eyelid enhancing. **D)** The largest excised masses (9×7 mm) of Case 2. Microscopically, the specimen contained chronic inflammation with foreign body lipogranuloma (H&E stain, ×100, ×400 with magnification).

A 30-year-old female visited our clinic for frequent upper eyelid swelling and a palpable medially located mass with lid lag and 2 mm lagophthalmos (Figure 2A). Although there were no definite findings, the patient wanted further evaluation and treatment. She said that the mass had recently enlarged and that laterally, a new small mass had developed. The patient had received rhinoplasty and autogenous fat injection in the forhead in March 2011 at a local plastic surgery clinic. Nine months after surgery, the above-mentioned symptoms developed. At first visit, MRI scans showed a highly enhanced mass in her left upper eyelid (Figure 2C). The surgically removed mass was 8~9 mm in diameter with chronic inflammation and foreign body lipogranuloma (Figure 2D). Six months after surgery, the mass and swelling had disappeared, and lid lag and lagophthalmos were also improved (Figure 2B).

The clinical characteristics of two patients were summarized (Table 1).

**Table 1:** The clinical characteristics of two patients

**Case**	**Symptoms**	**Surgery at plastic surgery / Symptom onset after 1st injection/ visit our clinic**	**Additional fat inection**	**Histopathogical findings**	**Postoperatively**
1	Lumps, Ptosis	Forehead augmentation **/** 7 months/ 13 months after 1^st^ inection	Yes*	Chronic inflammation with foamy histiocytic aggregation	No recur
					No lagophthalmos
					No lid lag
2	Lumps, Swelling	Forehead augmentation with rhinoplsty **/** 9 monnths / 12months after 1^st^ injection	No	Chronic inflammation with foreign body lipogranuloma	No recur Still remained lagophthalmos, lid lag

Yes* means the patient received additional fat injection using frozen storage autogenous fat tissue.

## Discussion

Autogenous fat is an excellent material for filling spot defects, such as depressed scars, and area defects, such as trough and sulcus deformities. Autogenous fat is easily accessible for harvesting and is available in adequate quantities for injection, even in lean patients. The fat is readily accepted at the implantation site due to its autogenous nature [1]. Facial injections, including periocular and paranasal injections of various substances, are becoming common as more procedures are performed under local anesthesia [2-4]. In young Asian subjects, autogenous fat injection for forehead augmentation is becoming increasingly popular to improve facial esthetics.

Complications of autogenous fat injection include a visible lump, the collection of fat at the injection site, an uneven skin surface, and allergic reactions to albumin or anesthetic agents. A rare, but potentially devastating, complication is embolization with loss of vision or stroke, which has been recently reported. The visual loss can result from retinal ischemia caused by occlusion of the ophthalmic and central arteries by fat emboli [5,6]. To our knowledge, however, there have been no previous reports of eyelid swelling with multiple lumps with or without blepharoptosis that regressed after surgical removal. The pathogenesis of eyelid swelling with small masses after autogenous fat injection for forehead augmentation could be explained by incorrect injection and/or anatomical variations of the forehead. One patient in this report received two injections of autogenous fat. The relatively short period between injections (in this patient, 4 weeks) suggest that overcorrection may have played a role in the development of complications. Furthermore, repeat injections generally used previously harvested fat stored in a freezer; and it is possible that this fat was less vital, less oxygenated, and did not survive well. The other patients who received a single injection received a relatively large volume of fat (0.3~0.5 ml), which may have been injected into the same area. This could become necrotic in less oxygenated areas and descend along the musculoaponeurotic system of the forehead to reach the superior orbital rim or lateral orbital rim, developing adhesions, fibrosis, and chronic inflammation, to ultimately form a cicatrix.

## Conclusions

Frequent eyelid swelling and multiple lumps with or without blepharoptosis are rare but potentially serious complications resulting from autogenous fat injection of the forehead. Here, we present the first report describing such adverse reactions and their treatment. Although blepharoptosis was not severe, if it progresses and does not improve with systemic anti-inflammatory medications, surgical intervention might be necessary, as it was in Case 1. Two patients were no longer suffered from frequent eyelid swelling after the masses were completely removed. Finally, if non-absorbable fatty tissue remains at the forehead, at the first injection site, it could move inferiorly and cause upper eyelid complications.

### Consent

Written informed consent was obtained from both patients for publication of this case report and any accompanying images. A copy of the written consent is available for review by the Editor in Chief of this journal.

## Competing interests

The authors declare that they have no competing interests.

## Author’s contributions

JSP, WKC, KSP and SWY treated the patient and in doing so acquired the case data; they were also involved with drafting of the manuscript. All authors read and approved the final manuscript.

## Pre-publication history

The pre-publication history for this paper can be accessed here:

http://www.biomedcentral.com/1471-2415/13/32/prepub
